# Supplementary material for: Resolving the full spectrum of human genome variation using Linked-Reads
Source: Genome Res. 2019 Apr;29(4):635–45. doi: 10.1101/gr.234443.118 (PMC6442396; doi:10.1101/gr.234443.118)
Supplement: Supplemental Material [file supp_gr.234443.118_Supplemental_longranger-2.2.zip › longranger-2.2.tar.bz2!/lr2.2-public/lib/stan/cmdstan/stan/doc/api/html/mean_8hpp_source.html]

Stan: src/stan/math/matrix/mean.hpp Source File


|  |  |
| --- | --- |
|  | Stan  2.5.0  probability, sampling & optimization |


- Main Page
- Related Pages
- Namespaces
- Classes
- Files

- File List
- File Members

All Classes Namespaces Files Functions Variables Typedefs Enumerations Enumerator Friends Macros Pages

- src
- stan
- math
- matrix

mean.hpp

Go to the documentation of this file.

1 #ifndef STAN\_\_MATH\_\_MATRIX\_\_MEAN\_HPP

2 #define STAN\_\_MATH\_\_MATRIX\_\_MEAN\_HPP

3

4 #include <vector>

5 #include <boost/math/tools/promotion.hpp>

6 #include <stan/math/matrix/Eigen.hpp>

7 #include <stan/math/error\_handling/matrix/check\_nonzero\_size.hpp>

8

9 namespace stan {

10  namespace math {

11

20  template <typename T>

21  inline

22  typename boost::math::tools::promote\_args<T>::type

23  mean(const std::vector<T>& v) {

24  stan::math::check\_nonzero\_size("mean(%1%)",v,"v", (double\*)0);

25  T sum(v[0]);

26  for (size\_t i = 1; i < v.size(); ++i)

27  sum += v[i];

28  return sum / v.size();

29  }

30

37  template <typename T, int R, int C>

38  inline

39  typename boost::math::tools::promote\_args<T>::type

40  mean(const Eigen::Matrix<T,R,C>& m) {

41  stan::math::check\_nonzero\_size("mean(%1%)",m,"m", (double\*)0);

42  return m.mean();

43  }

44

45  }

46 }

47 #endif

stan::math::mean

boost::math::tools::promote\_args< T >::type mean(const std::vector< T > &v)

Returns the sample mean (i.e., average) of the coefficients in the specified standard vector...

**Definition:** mean.hpp:23

Eigen.hpp

stan::math::sum

double sum(std::vector< double > &x)

**Definition:** sum.hpp:10

check\_nonzero\_size.hpp

stan::math::check\_nonzero\_size

bool check\_nonzero\_size(const char \*function, const T\_y &y, const char \*name, T\_result \*result)

Return true if the specified matrix/vector is of non-zero size.

**Definition:** check\_nonzero\_size.hpp:33


---

[ Stan Home Page ]

*© 2011–2014,
Stan Development Team.*
